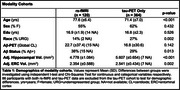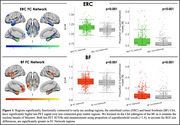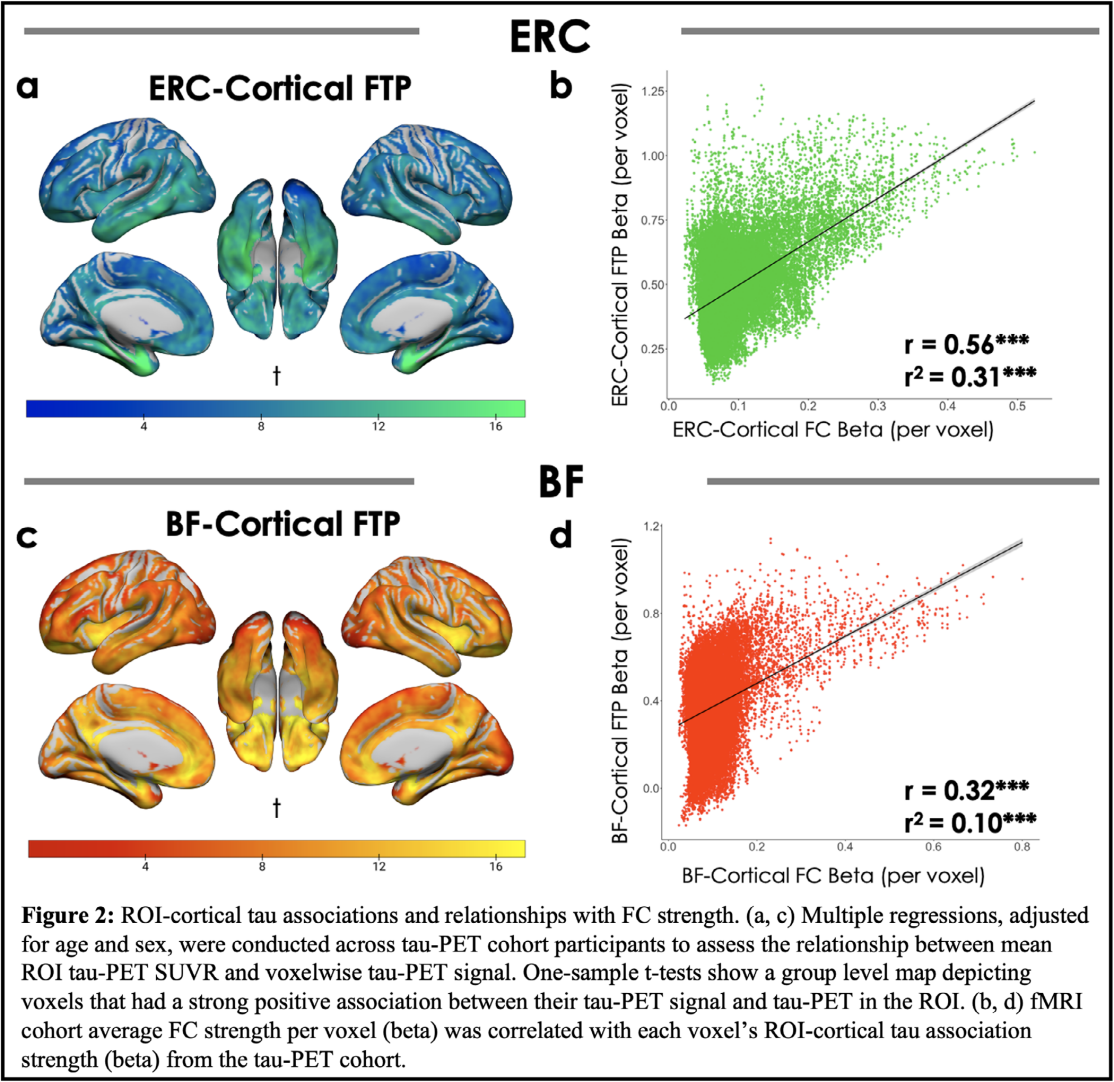# Basal forebrain and entorhinal cortex functional connectivity predicts regional tau burden in cognitively normal older adults

**DOI:** 10.1002/alz70862_109883

**Published:** 2025-12-23

**Authors:** Trevor A Chadwick, Corrina S. Fonseca, Jenna N. Adams, William J. Jagust, Theresa M. Harrison

**Affiliations:** ^1^ University of California, Berkeley, Berkeley, CA USA; ^2^ University of California, Irvine, Irvine, CA USA; ^3^ Lawrence Berkeley National Laboratory, Berkeley, CA USA

## Abstract

**Background:**

Pathological tau spreads trans‐synaptically in an activity‐dependent manner. Previous findings support that tau spread can be measured in vivo in regions functionally connected to the entorhinal cortex (ERC). We hypothesized that the functional connectivity (FC) strength of the basal forebrain (BF), another site of early tau deposition, would also predict patterns of tau spread.

**Method:**

We quantified flortaucipir tau‐PET scans from unimpaired older adults in ADNI (*n* = 351) and the Berkeley Aging Cohort Study (BACS; *n* = 99). For ERC and BF regions of interest, seed‐to‐voxel functional connectivity (FC) networks were generated using resting‐state fMRI in a partially overlapping BACS sample (*n* = 120) (Table 1). Outside‐network ROIs were created by subtracting the target FC network from a gray‐matter mask. Tau‐PET SUVR and the proportion of suprathreshold voxels (>1.4 SUVR) in FC network and outside‐network ROIs were compared with paired t‐tests. Voxel‐wise multiple regression analyses were used to measure the correlation between tau‐PET in the ERC or BF and the cortex. We used these maps to explore their relationship with seed‐to‐voxel FC strength.

**Result:**

FC of the ERC included the medial temporal, lateral temporal, and limbic regions, while FC of the BF included the insula, dorsal anterior cingulate, and limbic regions (Figure 1). Tau‐PET uptake was significantly greater in both the ERC and BF FC network compared to outside the network, using both SUVRs and proportion of suprathreshold voxels as tau measures. When comparing FC networks to the outside‐network ROIs, effects were greater for the ERC seed compared to BF (proportion of suprathreshold voxels: ERC d=0.85; BF d=0.49). In voxels across the cortex, the strength of FC to the ERC or BF was significantly correlated with the strength of cortical tau association to the ERC or BF (Figure 2).

**Conclusion:**

Significantly greater tau‐PET signal in the ERC and BF FC networks suggests that FC patterns of early tau accumulating regions predict pathological tau deposition across the cortex. The ERC seed provided stronger evidence for FC‐mediated tau spread compared to BF. Future research will investigate whether amyloid and APOE4 carrier status moderate the relationship between FC and tau spread from these early tau regions.